# Cortex Integrity Relevance in Muscle Synergies in Severe Chronic Stroke

**DOI:** 10.3389/fnhum.2014.00744

**Published:** 2014-09-23

**Authors:** Eliana García-Cossio, Doris Broetz, Niels Birbaumer, Ander Ramos-Murguialday

**Affiliations:** ^1^Institute of Medical Psychology and Behavioral Neurobiology, MEG Center, University of Tübingen, Tübingen, Germany; ^2^Ospedale San Camillo, Istituto di Ricovero e Cura a Carattere Scientifico, Venezia, Italy; ^3^German Center for Diabetes Research (DZDe.V.), Tübingen, Germany; ^4^TECNALIA, Health Technologies, San Sebastian, Spain

**Keywords:** muscle synergies, lesion location, neurorehabilitation, FMA, stroke

## Abstract

**Background:** Recent experimental evidence has indicated that the motor system coordinates muscle activations through a linear combination of muscle synergies that are specified at the spinal or brainstem networks level. After stroke upper limb impairment is characterized by abnormal patterns of muscle activations or synergies.

**Objective:** This study aimed at characterizing the muscle synergies in severely affected chronic stroke patients. Furthermore, the influence of integrity of the sensorimotor cortex on synergy modularity and its relation with motor impairment was evaluated.

**Methods:** Surface electromyography from 33 severely impaired chronic stroke patients was recorded during 6 bilateral movements. Muscle synergies were extracted and synergy patterns were correlated with motor impairment scales.

**Results:** Muscle synergies extracted revealed different physiological patterns dependent on the preservation of the sensorimotor cortex. Patients without intact sensorimotor cortex showed a high preservation of muscle synergies. On the contrary, patients with intact sensorimotor cortex showed poorer muscle synergies preservation and an increase in new generated synergies. Furthermore, the preservation of muscle synergies correlated positively with hand functionality in patients with intact sensorimotor cortex and subcortical lesions only.

**Conclusion:** Our results indicate that severely paralyzed chronic stroke patient with intact sensorimotor cortex might sculpt new synergy patterns as a response to maladaptive compensatory strategies.

## Introduction

Stroke survivors with upper limb (UL) motor impairment present an abnormal muscle activation pattern particularly at the level of hand muscles (Cauraugh et al., 2000; Langhorne et al., 2009).

Recent experimental evidence has indicated that the motor system may coordinate muscle activations through a linear combination of muscle synergies (muscle patterns) that are specified at the level of the spinal or brainstem networks (D’Avella et al., 2006; Bizzi et al., 2008). Cheung et al. (2009) suggested that descending signals from the motor cortical areas activate these networks, which in turn activate the motoneurons of a set of muscles with a particular muscle activation profile, i.e., different movements then emerge as the synergies are recruited to varying degrees (Cheung et al., 2009). After stroke cortical damage interferes with the flow of descending signals to the modular interneuronal structures of the spinal cord, which are responsible for activating groups of muscles as individual units (muscle synergies), and therefore, abnormal orchestration of synergies is present.

Recent findings showed on one hand that while in mildly impaired acute patients (*N* = 21) muscle synergies of the paralyzed UL were strikingly similar to those of the healthy one (despite remarkable differences in motor performance), subjects with severe motor impairment [fugl-Meyer-assessment (FMA) score ≤30] (*N* = 10), regardless of time since stroke and lesion location (cortical and/or subcortical lesions), presented much less similarity between the synergies of the two ULs (Cheung et al., 2012). The reduction in the number of muscle synergies in the paralyzed limb has been interpreted as a combination or merging of a number of synergies of the healthy limb onto one synergy of the paralyzed limb (Cheung et al., 2012). Moreover, this merging was found to correlate negatively with FMA, indicating that merging of specific muscle synergies can potentially lead to a reduction in the functionality of the UL. Merging of muscle synergies could be associated with the post-stroke “cocontractions” of muscles (Dewald et al., 1995), motor-module fusion of the affected lower limb of stroke patients (Clark et al., 2010), and the post-stroke couplings of shoulder and elbow actions (Dewald et al., 1995), which might account for a reduction of joint motion and hand functionality (Cheung et al., 2012).

On the other hand, in a subset of patients with chronic stroke (*N* = 11), a portion of the synergies in the paralyzed UL appeared to be divisions or fractionations of the synergies observed in the healthy side. This fractionation was correlated positively with chronicity (Cheung et al., 2012). Even though, these muscle synergy patterns (number of muscle synergies, merging, and fractionation) could be used as physiological markers of motor cortical damage, the mechanisms behind them and the possible brain structures involved at the cortical and subcortical level remained still unknown.

This study aim at characterizing the integrity of muscle synergy patterns in severe chronic stroke patients during different movements involving proximal and distal musculature and, specifically, at assessing (1) whether after entering in the chronic stage synergies from the healthy UL are preserved in the severely paralyzed UL as in the acute stage (Cheung et al., 2009), (2) whether the integrity of the sensorimotor cortex plays a role or not in the preservation, fractionation, or merging of synergies, and (3) how preservation of healthy UL synergies in the paralyzed limb relates to motor impairment.

## Materials and Methods

### Participants

Thirty-three chronic stroke patients (mean age 55, 12 female, and mean time after stroke 61.3 months) with subcortical only (*N* = 14) and mixed (cortical and subcortical) lesions (*N* = 19) were recruited via public information all over Germany. Magnetic resonance imaging (MRI) was used to verify lesion extent and location in every patient (Table 1; Table S1 in Supplementary Material). Patients were selected according to strict selection criteria, which included (1) no residual finger extension; (2) time since stroke at least 10 months (Ramos-Murguialday et al., 2013); (3) age between 18 and 80 years; (4) no psychiatric or neurological condition other than stroke; (5) no cerebellar lesion or bilateral motor deficit (see Table S1 in Supplementary Material); (6) no pregnancy; (7) no claustrophobia; (8) no epilepsy or medication for epilepsy during the last 6 months; (9) eligibility to undergo MRI; and (10) ability to understand and follow instructions [mini-mental state (MMS) score above 21] [for more details see Ramos-Murguialday et al. (2013)].

**Table 1:** **Patients lesion, synergy information, and functional state of paralyzed upper limb**.

	Patient	Number of recorded electrodes	Paralyzed limb		Healthy limb		No. shared synergies	Merging index	Fractionation index	FMA hand/ finger	ASHW
			No. optimal synergies	% *r*^2^	No. optimal synergies	% *r*^2^	
Mixed	1	7	4	96.62	1	93.12	0.95	0	0.00	9	11
	2	6	1	95.18	2	97.49	0.15	0	0.00	3	15
	3	8	2	97.27	3	96.27	1.45	1	1.92	3	2
	4	8	4	95.98	5	96.53	1.45	1	2.74	3	6
	5	8	5	96.87	4	97.56	1.15	2	0.00	3	3
	6	8	2	96.70	5	97.05	1.55	2	1.91	2	2
	7	8	3	92.57	4	96.22	1.30	1	0.00	1	21
	8	8	4	96.34	2	97.30	0.80	0	1.92	4	6
	9	6	4	96.94	2	96.95	0.75	0	0.00	8	18
	10	8	1	95.50	1	92.74	0.10	0	1.56	0	5
	11	8	2	97.12	2	97.05	0.05	1	0.00	2	0
	12	8	3	95.36	4	97.13	1.50	3	1.61	2	10
	13	8	4	96.67	4	95.08	1.60	2	0.00	2	8
	14	8	3	92.61	4	96.74	2.15	2	1.82	2	6
	15	8	4	94.39	5	97.13	1.05	1	0.00	4	7
	16	8	2	96.04	3	96.70	0.60	1	0.00	3	13
	17	8	4	96.36	4	96.51	0.75	3	0.00	2	10
	18	8	2	96.36	3	96.13	1.35	2	0.00	3	3
	19	8	2	96.94	3	97.50	1.05	1	0.00	3	17
Subcortical	20	8	2	97.01	4	96.40	0.40	0	0.00	2	8
	21	8	1	98.18	2	96.67	0.90	0	1.65	6	21
	22	8	4	96.75	5	96.31	0.45	2	0.00	2	3
	23	8	1	97.54	2	94.06	0.90	1	1.87	2	26
	24	8	5	95.99	4	96.10	0.75	2	0.00	1	5
	25	8	4	94.34	4	96.83	0.50	2	1.66	0	3
	26	8	2	95.89	3	97.46	0.45	1	0.00	0	11
	27	8	3	95.70	5	95.18	1.50	1	0.00	6	2
	28	8	1	93.02	2	97.17	0.35	1	2.74	0	29
	29	8	4	97.13	2	96.56	0.85	2	0.00	3	2
	30	8	1	97.13	3	97.23	1.00	1	0.00	11	9
	31	8	4	97.13	3	95.91	0.60	1	0.00	6	4
	32	8	1	97.21	3	97.20	0.25	0	0.00	3	13
	33	7	1	93.96	2	91.91	0.90	1	0.00	11	9

*M, mixed lesion; S, subcortical lesion; ASHW, Ashworth scale; No., number*.

The study was approved by the ethics committee of the Faculty of Medicine of the University of Tübingen and all the patients gave informed consent.

### Assessment of motor status of the paralyzed UL

The motor status of the paralyzed UL for each patient was evaluated using two scales: (1) the modified UL for hand/finger motor scores from the fugl-Meyer assessment scale (hFMA) (with a maximal score of 24 points); (2) the Ashworth scale for measuring muscle spasticity (with a maximal score of 56) (Table 1). Items assessing upper extremity sensation and pain, coordination, speed, and reflexes were excluded from FMA (Crow and Harmeling-van der Wel, 2008).

### EMG recordings and task

**Figure 1:**
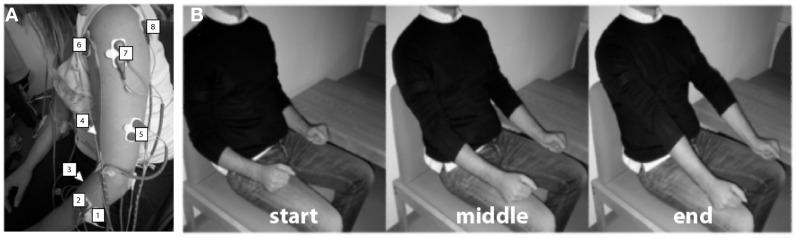
**Experimental procedure**. **(A)** Electrode array. Eight bipolar EMG electrodes were positioned on (1) extensor carpi ulnaris, (2) extensor digitorum, (3) flexor carpi radialis, palmaris longus, flexor carpi ulnaris (flexion), (4) long head of the biceps (flexion), (5) the external head of the triceps, (6) anterior portion of deltoid muscle, (7) lateral portion of deltoid muscle, and (8) posterior portion of deltoid over the teres minor and infraspinatus muscles. **(B)** Example of visual instructions illustrating elbow extension. Three different pictures were presented to the patients during the task instruction and execution period, each one representing the starting point of the task [**(B)**-left], the intermediate [**(B)**-middle], and the end position [**(B)**-right].

The surface EMG activity was recorded from both UL using bipolar electrodes placed on (1) extensor carpi ulnaris, (2) extensor digitorum, (3) flexor carpi radialis, palmaris longus, flexor carpi ulnaris, (4) long head of the biceps, (5) the external head of the triceps, (6) anterior portion of deltoid muscle, (7) lateral portion of deltoid muscle, and (8) posterior portion of deltoid over the teres minor and infraspinatus muscles (Figure 1A), during different movements.

Patients were asked to perform six different arm and hand movements: (1) flexion of the upper arm, (2) elbow rotation, (3) extension of the elbow, (4) supination, (5) wrist extension, and (6) finger extension (Figure 1B). Specifically, these movements were related to the items to evaluate arm FMA (shoulder flexion 0°–90°, shoulder abduction 0°–90°, and pro-supination elbow in flexion and in extension) and hand FMA (wrist extension elbow at 90° and finger extension). During each movement, the patients were presented with a correspondent classical music piece (different for each movement) increasing in volume during the entire 12 s of each trial (instructions + ready + movement). This was used as a rhythmic motivation and concentration tool. A silent inter-trial period between 4 and 7 s was used to allow the patients to return to the resting/start position (hands resting on their lap).

Patients were instructed to perform each movement with both arms simultaneously after a “Go” cue during 6 s maintaining their gaze on the screen. The patients had to try to perform these movements with the affected and the unaffected ULs simultaneously. Compensatory movements were discouraged. The experiment was divided in blocks. One block contained 60 trials, 10 for each of the 6 different movements. On average patients underwent between 4 and 6 blocks with a total of 40–60 trials per condition.

### EMG pre-processing

The EMG data were band pass filtered between 50 and 500 Hz. The line noise was rejected using a notch filter at 50 Hz. After filtering, the data were normalized using the inter-trial interval (as a rest condition) and rectified. The envelope of the signal was calculated using a low-pass filter of 2 Hz and afterwards re-sampled to 5 samples/s. Trials containing contaminated data (such as no movement, bad impedance, interference of the EMG cables, EMG overshooting, and among others) were identified by an expert and disregarded for the subsequent analysis. We created a pooled data set that included all six performed movements for muscle synergy extraction.

### Extracting muscle synergies

Muscle synergies were extracted from the pre-processed EMG recordings from all movements together using a non-negative matrix factorization (NMF) algorithm (Lee and Seung, 2001).

**Figure 2:**
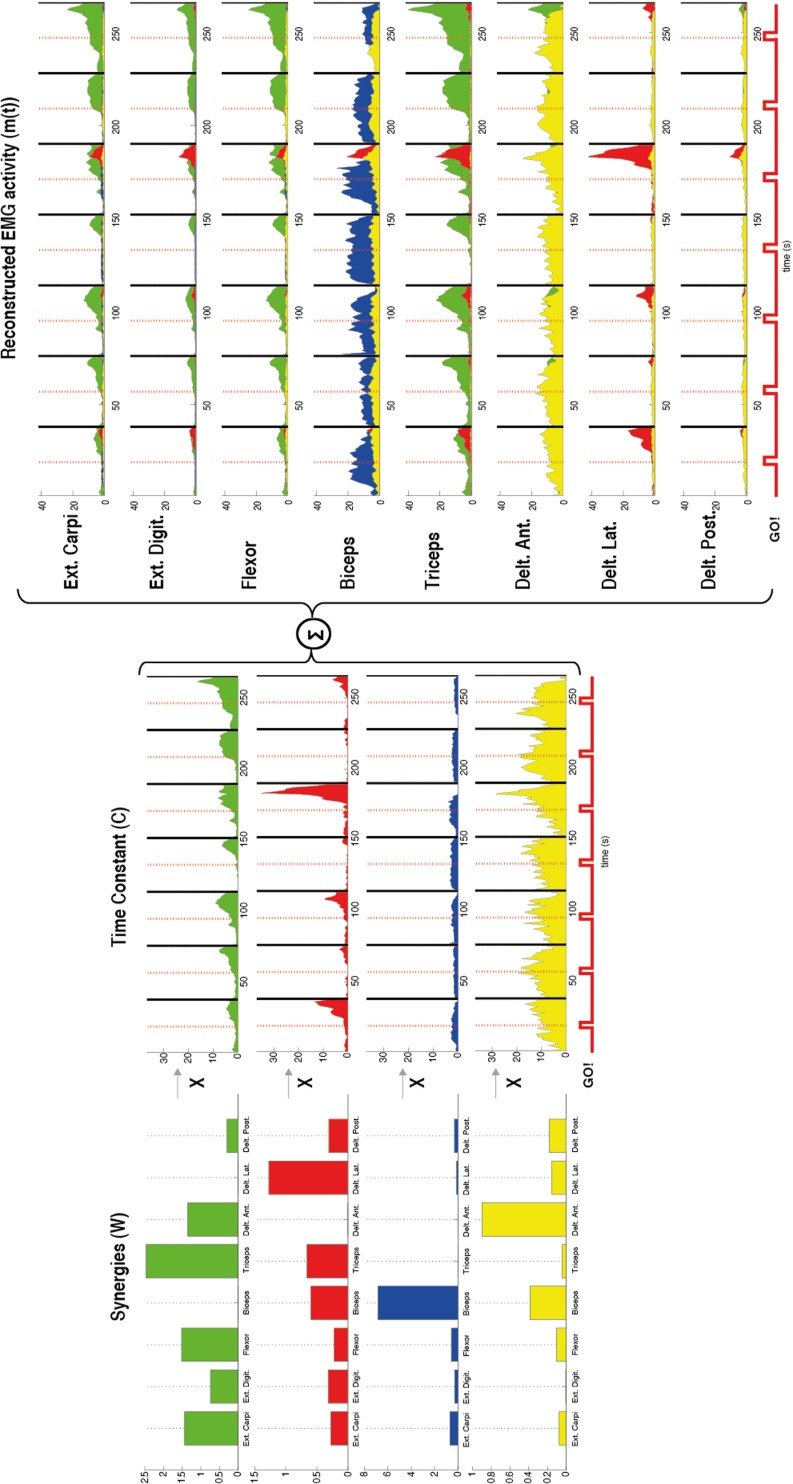
**Representation of the muscle synergies calculated by using non-negative matrix factorization in one patient during flexion of the upper arm**. The factorization algorithm was applied in this case by using four synergies (*n* = 4). For each synergy (W) (left) the contribution of each muscle [external carpi ulnaris (Ext. Carpi), external digitorium (Ext. Digit.), flexor, biceps, triceps, anterior (Delt. Ant.), lateral (Delt. Lat.), and posterior portion of deltoids (Delt. Post.)] is represented by the amplitude of each bar. Different muscles with different gains contributed to each synergy. For example, the first synergy in green has contribution of almost all the muscles except the biceps and lateral deltoid. Furthermore, a time constant (C) (middle) for each synergy (W) was also calculated by the factorization algorithm. These constants represent how much each synergy contributes along the time to the task. After the synergies (W) and the time constants (C) were calculated the EMG was reconstructed by the sum of the products between these two variables (right). The reconstructed EMG [*m*(*t*)] shows for each recorded muscle the time course of the EMG activity and in different colors the contribution of each synergy to the task. A stronger EMG activity is seen after each Go! Cue, which indicates the beginning of the movement in each trial.

The muscle activation vector *m*(*t*) (with same units of EMG amplitude) is represented by the following equation:

$$m\left(t\right)=\sum_{i-1}^{n}C_{i}\left(t\right)W_{i}$$

where *C_i_*(*t*) are time-varying coefficients, *W_i_* are fixed element muscle vectors (synergies) with the elements representing the relative activation of each muscle, and *n* is the number of synergies. A representation of this factorization process is shown in Figure 2.

### Synergy features

#### Optimal number of synergies

We applied the NMF algorithm (Lee and Seung, 2001) with *n* increasing from one to eight (total number of recording electrodes). For each patient we tested the goodness of the EMG reconstruction according to the number of synergies included (*n*) [i.e., how similar was *m*(*t*) to the original EMG data] by choosing randomly for each *n* half of the EMG trials for extracting the synergies (W) and the other half for testing them.

The goodness of the fitting, measured by *r*^2^, was then plotted as a function of the number of synergies included in the model. This procedure was also performed for shuffled EMG data (generated by shuffling randomly the EMG data across muscles and time). The *r*^2^ curve of the shuffle data represents the chance level showing an almost constant slope from 0 to 1. This process was repeated iteratively using a 20-fold cross-validation, in order to test robustness of the algorithm (e.g., Figure S1 in Supplementary Material) (Cheung et al., 2005). The *optimal number of synergies* (i.e., representing the minimum number of synergies required for adequate reconstruction of the EMGs) was calculated by defining the critical point of the *r*^2^ curve where the curve’s slope decreases <0.0005 and its respective optimal synergies were extracted and used in subsequent analysis.

#### Synergy similarity among upper limbs

The optimal synergies from both ULs were compared against each other one by one by calculating the similarity between them assessed by the scalar product (with a scalar product of 1 representing full matching and a scalar product of 0 indicating no similarity at all). The pairs of synergies were selected according to the magnitude of the scalar product and organized from the most similar (scalar product close to 1) to the less similar (scalar product close to 0). This procedure was done also for non-structured synergies (generated by shuffling randomly the vector W). In order to calculate the number of shared synergies, a *threshold for similarity* was obtained by corrupting synergies [generated by shuffling randomly the weight vector (W) containing the contribution of each muscle to each synergy] for both ULs and calculating the maximum scalar product among them. If the scalar product of the affected and unaffected synergies was larger than the *threshold for similarity*, this particular pair of synergies was considered as a shared synergy. This procedure was repeated iteratively using 20-fold cross-validation. The number of shared synergies was calculated for each patient by averaging the number of shared synergies across the 20-folds (Table 1).

#### Merging and fractionation

Furthermore, we investigated whether the observed muscle synergies in the paralyzed limb could be explained as linear combination of multiple synergies from the healthy limb (synergy merging) or whether some paralyzed UL synergies could be explained as fractionation or division of a healthy UL synergy (synergy fractionation). Merging and fractionation patterns of muscle synergies were calculated according to Cheung et al. (2012).

The merging index was defined as the number of muscle synergies from the paralyzed UL that resulted from merging of two or more synergies from the healthy UL. The scalar product between the reconstructed (by merging) paralyzed UL synergy and the real paralyzed UL synergy was calculated, and only healthy UL synergy merging was counted if the scalar product was above 0.75. The fractionation index was defined as the mean number of muscle synergies of the paralyzed UL that resulted from the fractionation of a paralyzed UL synergy. The same constrain for the scalar product was applied.

### Clustering synergies

Optimal synergies derived from the optimal number of synergies from each patient were pooled together for each UL separately and categorized into clusters (different group of synergies with a common muscle activation pattern) for a global synergy comparison between ULs. Since it has been demonstrated that synergies are preserved and are very similar within patients, we expected to see no differences between ULs when clustering synergies.

For this purpose, we implemented a hierarchical cluster analysis available in the Statistical Toolbox of Matlab, which consist of (1) finding the similarity or dissimilarity between every pair of synergies in the data set by calculating the Minkowski distance between objects using the *pdist* function; (2) grouping the objects into a binary, hierarchical cluster tree by linking pairs of synergies that are in close proximity using the *linkage* function (Ward option); and (3) determining where to cut the hierarchical tree into clusters by using the *cluster* function to prune branches off the bottom of the hierarchical tree and assign all the objects below each cut to a single cluster, which creates a partition of the data with the main synergy representation across patients.

**Figure 3:**
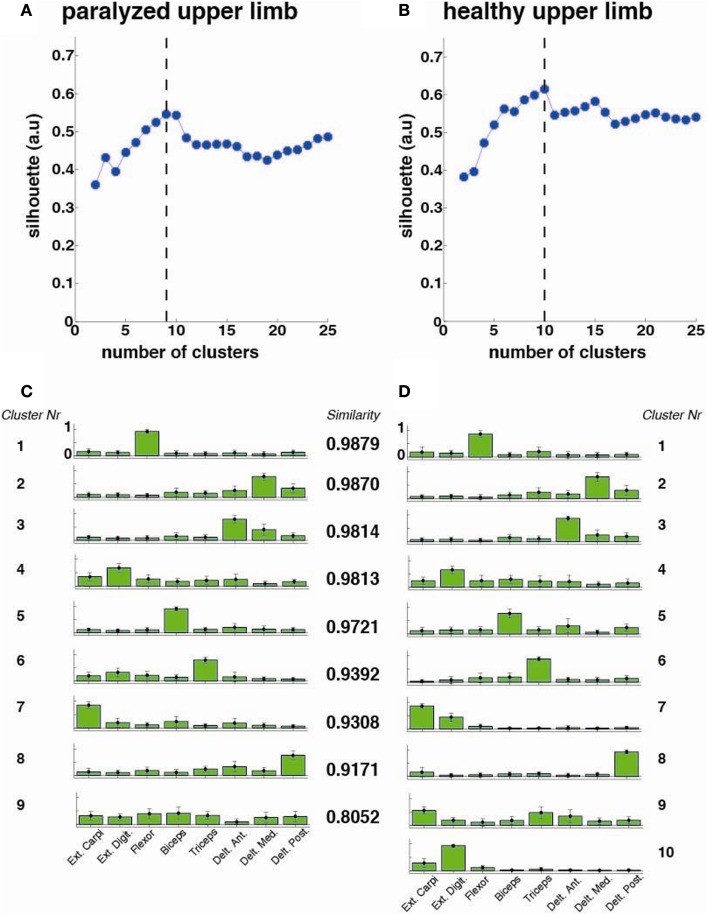
**Cluster analysis for all patients (*N* = 33)**. The silhouette value for the **(A)** paralyzed and **(B)** healthy upper limb was calculated. The dashed line represents the optimal number of clusters, which were extracted and plotted for the paralyzed **(C)** and healthy **(D)** upper limbs. Each cluster is represented by the normalized contribution (mean ± SD) (from 0 to 1, where 1 is the maximum contribution) of each recorded muscle across patients (green bars): (1) extensor carpi ulnaris (Ext. Carpi), (2) extensor digitorum (Ext. Dig.), (3) flexor carpi radialis, palmaris longus, flexor carpi ulnaris (flexor), (4) long head of the biceps, (5) the external head of the triceps, (6) anterior portion of deltoid muscle (Delt. Ant.), (7) lateral portion of deltoid muscle (Delt. Lat.), and (8) posterior portion of deltoid over the teres minor and infraspinatus muscles (Delt. Post). The *similarity* between synergies (clusters) from the healthy and paralyzed upper limb was measured by the scalar product (a *similarity* value of 1 means perfect match between them).

The optimal number of synergy-clusters that represented the best optimal synergies for all patients was determined based on the silhouette index, which evaluates the goodness of a clustering structure (Wang et al., 2009). The silhouette index reflects the compactness and separation of clusters and it is calculated based on the average distance within and between clusters, so a high number (close to 1) of silhouette reflects a good clustering. We calculated and plotted the silhouette as a function of the number of clusters (from 2 to 25 clusters) included in the analysis. The optimal number of clusters was calculated by defining the maximum point of the silhouette index’s curve (see Figure 3 top row).

In order to observe particular effects of sensorimotor cortex integrity on synergy modularity, we performed the same analysis but this time separating the patients according to the lesion location in subcortical (intact sensorimotor cortex) (hFMA = 4 ± 3.72) and mixed (cortical and subcortical) (hFMA = 3 ± 2.13) lesion groups. Lesions at a cortical level included lesions in the sensorimotor cortex only. This separation was carried out by an experienced radiologist using the patients T1 MR images (Table S1 in Supplementary Material).

### Statistical analysis

All data were reported as mean values ±SD when indicated. Because FMA scores are ordinal we used the Spearman’s rank correlation (a non-parametric version of the Pearson correlation). Statistical evaluations on synergy patterns were performed using *T*-test with 95% CI.

## Results

### Number of muscle synergies and synergy similarity

Reconstruction of EMG data using the optimal number of synergies was measured by the *r*^2^ value, which was for both ULs larger than 96% across patients (paralyzed UL: *r*^2^ = 96.02 ± 1.42; healthy UL: *r*^2^ = 96.25 ± 1.40) (Table 1) (Figure S1 in Supplementary Material), showing robustness of the NMF algorithm.

**Figure 4:**
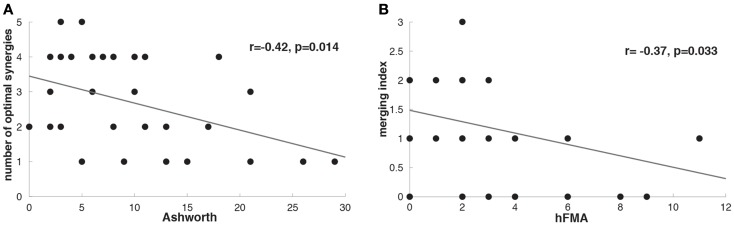
**(A)** Correlation between Ashworth scale and number of optimal affected synergies (*r* = −0.42, *p* = 0.014). **(B)** Correlation between hFMA (hand Fugl-Meyer Assessment scores) and merging index (*r* = −0.37, *p* = 0.033).

The number of optimal synergies calculated across patients in the paralyzed limb was slightly reduced in comparison to the healthy one (*t* = −1.17 *p* = 0.06, paralyzed UL = 2.73 ± 1.33, healthy UL = 3.18 ± 1.18). We tested if this change could be due to spasticity and we observed indeed that the number of optimal synergies from the paralyzed limb correlated negatively with spasticity [i.e., the higher the spasticity (higher Ashworth score) the lower the number of optimal synergies] (*n* = 33, *r* = −0.42, *p* = 0.014) (Figure 4A) indicating a decrease of complexity in the EMG data with spasticity.

### Clustering synergies

The cluster analysis across all patients revealed a high similarity (similarity >0.75) between paralyzed and healthy ULs synergies as reported before for mildly stroke patients (Cheung et al., 2009) (Figure 3).

**Figure 5:**
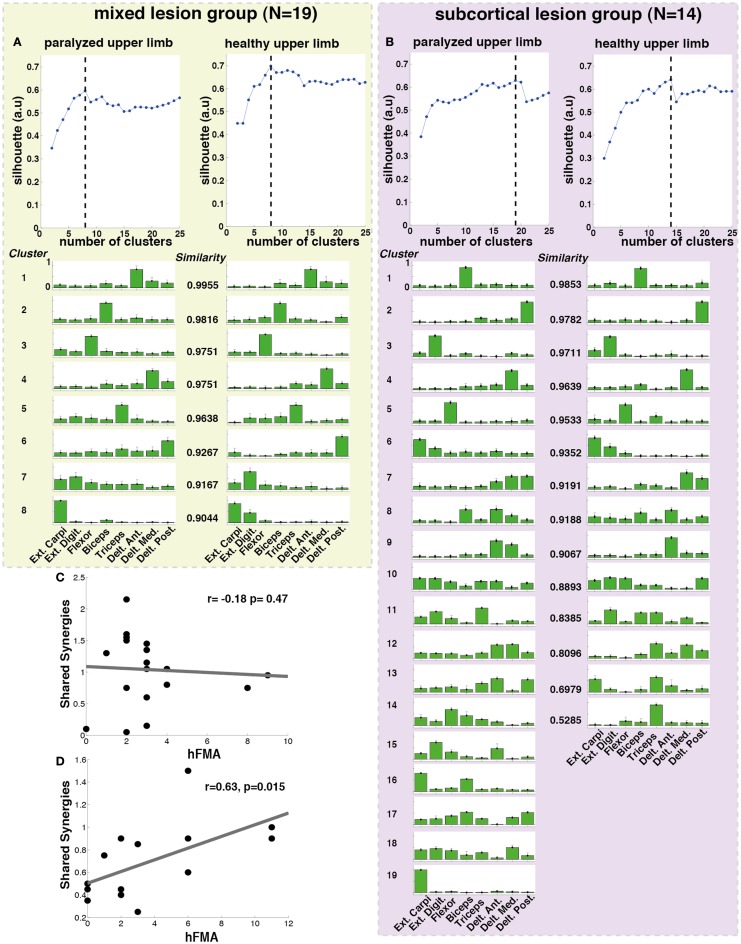
**Lesion location related cluster analysis**. The analysis was performed independently for the mixed lesion group of patients [green box **(A)** paralyzed and healthy limb] and the subcortical lesion group [purple box **(B)** paralyzed and healthy limb]. On the top of each column **(A,B)**, the silhouette (blue) index representing the goodness of the clustering is illustrated. The dashed black lines indicate the optimal number of clusters. Each cluster is represented by the normalized contribution (from 0 to 1, where 1 is the maximum contribution) of each electrode across patients (green bars) placed on top of (1) extensor carpi ulnaris (Ext. Carpi), (2) extensor digitorum (Ext. Dig.), (3) flexor carpi radialis, palmaris longus, flexor carpi ulnaris (flexor), (4) long head of the biceps, (5) external head of the triceps, (6) anterior portion of deltoid muscle (Delt. Ant.), (7) lateral portion of deltoid muscle (Delt. Lat.), and (8) posterior portion of deltoid over the teres minor and infraspinatus muscles (Delt. Post). The *similarity* between synergies (clusters) from the healthy and paralyzed UL was measured by the scalar product (a *similarity* value of 1 means perfect match between them). **(C)** No significant correlation was found between hFMA (hand Fugl-Meyer) and number of shared synergies (*n* = 19, *r* = −0.18 *p* = 0.478) in patients with mixed lesion, **(D)** while a significant positive correlation between hFMA and number of shared synergies (*n* = 14, *r* = 0.63, *p* = 0.015) was found in patients with subcortical lesion only.

**Figure 6:**
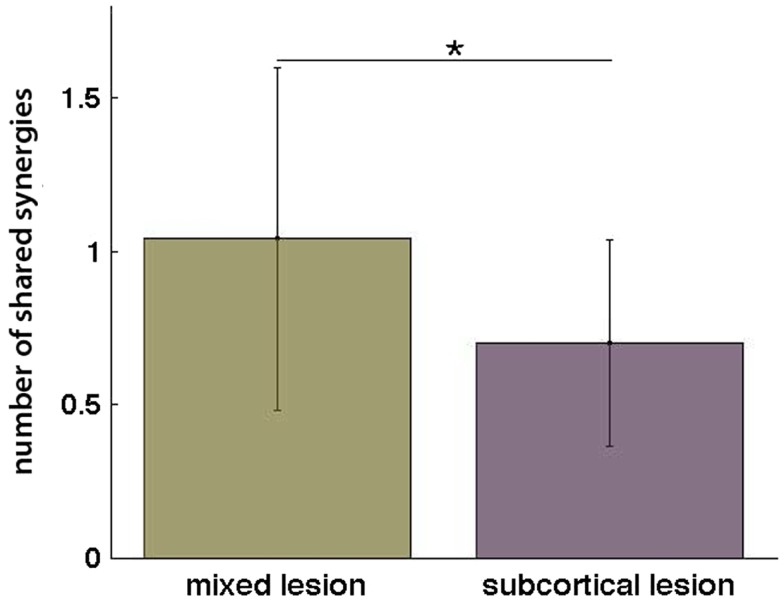
**Number of shared synergies in the mixed lesion and subcortical lesion group (mean ± SD)**. *p = 0.05.

Additionally, when the lesion group division was done, we found that muscle synergies from the healthy UL were highly preserved in the paralyzed UL in the group of patients with mixed lesion (similarity >0.75) (Figure 5A) and in a lower degree in the patients with subcortical lesion only (Figure 5B). Moreover, we found a remarkable increase in the number of muscle synergies extracted from the cluster analysis of the paralyzed UL across patients in the subcortical lesion group (healthy UL = 14, paralyzed UL = 19) (Figure 5B) in comparison to the mixed lesion group (healthy UL = 8, paralyzed UL = 8) (Figure 5A). This indicates an increase in the number of necessary clusters to correctly represent the data set of the subcortical group, which might indicate a broader spectrum of movement strategies across patients with subcortical lesion only. Furthermore, we found a significant reduction in the number of shared synergies in the subcortical compared to the mixed lesion group of patients (*p* = 0.05) (Figure 6). In the subcortical group, some muscle synergies of the paralyzed limb were modified in comparison to the matched healthy limb muscle synergy in 2 of 19 clustered synergies (similarity <0.75) (Figure 5B) and 5 paralyzed UL synergies were not matched with any healthy UL synergy. This synergy modification could represent new, merged, or fractionated synergies as a result of preserved cortical structures (sensorimotor cortex), which might attempt to modify synergies modules as a product of neurophysiological compensatory.

Furthermore, we have found that in patients with subcortical lesion only, the number of shared synergies correlated positively with motor functionality (negatively with motor impairment) of the UL (hFMA: *n* = 14, *r* = 0.63, *p* = 0.015) (Figure 5D). The higher the number of shared synergies between the paralyzed and healthy ULs the lower motor impairment in patients with subcortical lesion. No significant correlation between the number of shared synergies and hFMA was found in the mixed lesion group of patients (hFMA: *n* = 19, *r* = −0.178, *p* = 0.47) (Figure 5C).

### Merging and fractionations

After identifying that the number of optimal synergies in the paralyzed limb was slightly reduced in comparison to the healthy one (see Number of Muscle Synergies and Synergy Similarity) we have try to find whether this difference in data dimensionality was attributed to a merging of healthy UL muscle synergies into a paralyzed UL synergy. We have found that in 25 patients merging of healthy UL muscle synergies into paralyzed UL synergies was present (Table 1). Furthermore, we have also investigated whether in some of the patients we could see instead of a merging pattern a fractionation of a healthy UL synergy into some paralyzed UL muscle synergies. We found that only in 11 patients a fractionation pattern was present (Table 1). Therefore, in relation to fractionation, merging of healthy UL synergies into one paralyzed UL synergy was a predominant pattern in patients with severe impairment in the hand, confirming previous observations (Cheung et al., 2012). Furthermore, we have found that higher merging lead to more reduced hand functionality (hFMA) (*n* = 33, *r* = −0.37, *p* = 0.033) (Figure 4B) (Cheung et al., 2012), indicating that the more severe the impairment in the hand the more likely a merging of multiple healthy UL synergies into one paralyzed UL synergy appeared.

Additionally, in order to explain the reduction in the number of shared synergies of the subcortical group in comparison to the mixed lesion group of patients, we have calculated among them the difference in merging and fractionation indexes. Surprisingly, we did not find any significant difference between groups for merging (*t* = 0.45 *p* = 0.66) or fractionation (*t* = 0.42 *p* = 0.68), indicating that other mechanisms (i.e., loss or generation of new synergies) due probably to preserved cortical structures and compensatory efforts might have been involved affecting synergy modules in the subcortical lesion group.

## Discussion

Muscle synergy information extracted from the EMG of severe chronic stroke patients revealed different physiological patterns for patients with and without preserved sensorimotor cortex.

On one hand, synergy information extracted by the cluster analysis revealed that in severely impaired chronic stroke patients, muscle synergies from the healthy UL were mostly preserved in the paralyzed UL. However, muscle synergy patterns changed when the sensorimotor cortex was intact, resulting in a significant increase in muscle synergies in the paralyzed UL and in a reduction in synergy similarity among limbs. Although previous studies (Cheung et al., 2012) suggested that a reduction in synergy similarity could be explained as a merging pattern of healthy UL synergies into paralyzed UL ones, we could not explain the difference in synergy similarity between the mixed and purely subcortical lesion group of patients as a difference in the merging index. Furthermore, the increment in the number of clusters necessary to correctly represent the data set of the subcortical in comparison to the mixed lesion group might be associated with a broader spectrum of movement strategies that patients with preserved sensorimotor cortex and subcortical lesion only developed after stroke. Therefore, the difference in synergy similarity and the number of muscle synergies between patients with mixed lesion and subcortical lesion only might indicate that other processes like neural changes at the cortical level (Yao et al., 2009) might be involved in the generation of new muscle synergies.

We have found that merging of healthy UL synergies into paralyzed UL ones was present in the majority of our severe chronic stroke patients (75.5%), confirming the appearance of this synergistic pattern in the severely impaired state as previously suggested (Cheung et al., 2012). Furthermore, we observed that the merging of healthy UL synergies correlated significantly with a higher impairment in the paretic UL. In line with these results, it has been suggested that merging could be attributed to stereotypical movement patterns described in stroke as coupling of shoulder and elbow (Dewald et al., 1995), which can account for reduction in the range of joint motion and therefore in the UL functionality. Additionally, we have observed that the more spastic the paralyzed limb the less muscle synergies needed for a good reconstruction of the EMG data, indicating a limitation in movement variety, i.e., less mobility, lower movement complexity, and fewer synergies needed.

In higher primates and humans, there are two subdivisions of the primary motor cortex: a rostral, phylogenetically older region that contains descending efferents destined to the spinal interneurons, and a caudal, phylogenetically younger region that contains corticomotoneuronal (CM) cells with monosynaptic innervations to the motoneurons of individual shoulder, elbow, and finger muscles (Rathelot and Strick, 2009). It is plausible that while the “old” motor cortex contributes to motor output by providing activation drives for the spinal modules, the “young” motor cortex further sculpts the activations of specific muscles by bypassing the spinal mechanisms through the CM cells. The overlap and intermingling of CM cells for different hand muscles enables M1 to create a wide variety of muscle synergies (Rathelot and Strick, 2006). From this point of view, after stroke the preservation of the motor cortex might play an important role in activating neurophysiological compensatory mechanisms to overcome the motor deficit, which could result in the establishment of new synergy patterns. However, these compensatory neuroplastic mechanisms may or may not contribute to the functional motor recovery of the paralyzed joints. For instance, we have found that patients with high similarity of muscle synergies among ULs presented less hand motor impairment in the group of patients with subcortical lesion only. The latter indicates that intact neuroplastic compensatory cortical mechanisms, which are supposedly involved in the generation of new muscle synergies, might result in maladaptation. The motor cortex could modify or sculpt new “wrong” synergies as a result of a lack of feedback (propioception) and reward and the appearance of maladaptive compensatory strategies (Cirstea and Levin, 2000). Our results indicate that after stroke the motor cortex represents an important pillar for integrating new muscle synergies (Rathelot and Strick, 2009) into the existing repertoire of synergies defined at the level of brain stem and spinal cord (Bizzi et al., 2008; Cheung et al., 2009). Stroke patients with preserved sensorimotor cortex might benefit from this process when directed toward motor recovery. Previous work (Ameli et al., 2009) has highlighted the relevance of the motor cortex in motor recovery after application of facilitatory non-invasive brain stimulation to the lesioned hemisphere. Patients with intact cortical networks were found to improve after non-invasive brain stimulation, whereas those patients featuring cortical lesions did not respond or even deteriorated in terms of motor functions of the paretic hand (Ameli et al., 2009). Therefore, understanding the mechanisms of action of the residual brain architecture of the patient will be crucial for implementation of future motor treatments (Riley et al., 2011). Rehabilitation strategies based on biofeedback (Basmajian, 1988) built upon synergy models might represent a promising tool to enhance functional motor recovery after stroke, as it has been successfully shown in chronic stroke patients using its variety neurofeedback in a brain machine interface scenario (Prasad et al., 2010; Ramos-Murguialday et al., 2013).

Future studies should consider the implementation of high-density EMG in order to increase the number of muscles recorded and the sensitivity to residual UL muscle activity.

## Conclusion

After stroke the sensorimotor cortex represents an important pillar for integrating new muscle synergies (Rathelot and Strick, 2009) into the existing repertoire of synergies defined at the level of brain stem and spinal cord (Bizzi et al., 2008; Cheung et al., 2009). Therefore, patients with intact sensorimotor cortex might have the required cortical plasticity to incorporate simple activation patterns involving different muscles (muscle synergy) in their movement repertoires throughout simple massive repetitive training. Thus, as motor training for as little as 6 weeks can induce changes in white matter (Scholz et al., 2009), muscle synergy-based robot training may encourage the development of new muscle synergies meaningful for rehabilitation.

## Conflict of Interest Statement

The authors declare that the research was conducted in the absence of any commercial or financial relationships that could be construed as a potential conflict of interest.

## Supplementary Material

The Supplementary Material for this article can be found online at http://www.frontiersin.org/Journal/10.3389/fnhum.2014.00744/abstract
