# Supplementary material for: Cortex Integrity Relevance in Muscle Synergies in Severe Chronic Stroke
Source: Front Hum Neurosci. 2014 Sep 23;8:744. doi: 10.3389/fnhum.2014.00744 (PMC4172028; doi:10.3389/fnhum.2014.00744)
Supplement: Supplementary file 1 [file Table1.PDF]

Supplementary Table 1. Demography data.

Code	Lesion	Sex	Age	Time since stroke (months)	Lesion location
1	Mx	F	31	15	Mixed lesion:cortical stroke affecting the frontal- and parietal lobe (pre- and postcentral gyrus).Corona radiata, capsula externa affected. Hypodense white matter of the frontal and parietal lobe.
2	Mx	F	65	131	Mixed lesion:Frontal and parietal lobe. Pre-postcentral gyrus.
3	Mx	M	60	14	Mixed lesion: parietal and occipital lobe and the adjacent white matter, multiple necrotic vesicles in the parietal lobe underneath the post-central and pre-central gyrus, gray matter of the post- and precentral gyrus partly intact
4	Mx	F	66	23	Mixed lesion. Frontal and parietal lobe. Superior, medial, middle and inferior frontal gyrus; pre and postcentral gyrus.
5	Mx	M	58	28	Mixed lesion. Frontal, parietal and temporal lobe. Middle and inferior frontal gyrus; pre and postcentral gyrus; supramarginal gyrus; angular gyrus. Trunk of corpus callosum, corona radiata, caudate nucleus, claustrum, putamen, CI, genu, thalamus, external capsule, insula.
6	Mx	F	54	10	Mixed lesion. Frontal, parietal and temporal lobe. Inferior frontal gyrus; pre and postcentral gyrus; middle temporal gyrus. Corona radiata, CI, genu, external capsule, putamen, thalamus.
7	Mx	F	73	23	Mixed lesion. Frontal, parietal and temporal lobe. Superior, medial, middle and inferior frontal gyrus; pre and postcentral gyrus. Corona radiata,thalamus, putamen, posterior CI partially, genu, external

					capsule, trunk of corpus callosum.
8	Mx	F	36	16	Mixed lesion. Frontal and parietal lobe. Middle and inferior frontal gyrus; postcentral gyrus. Corona radiata, caudate nucleus, external capsule, CI, genu, thalamus, putamen, insula.
9	Mx	M	51	139	Mixed lesion. Frontal and parietal lobe. Encephalomalacia in the frontal and parietal lobe. Middle and inferior frontal gyrus; pre and postcentral gyrus; supramarginal gyrus.
10	Mx	M	51	16	Mixed lesion. Frontal and parietal lobe and adjacent white matter with multiple necrotic. Middle and inferior frontal gyrus; pre and postcentral gyrus; middle temporal gyrus.
11	Mx	M	69	89	Mixed lesion. Frontal, parietal and temporal lobe. Middle and inferior frontal gyrus; pre and postcentral gyrus; supramarginal gyrus; middle and inferior temporal gyrus. Corona radiata, head of caudate nucleus, CI, genu, external capsule, claustrum, putamen, truck of corpus callosum, insula, thalamus.
12	Mx	F	35	28	Mixed lesion. Frontal, parietal and temporal lobe. Inferior frontal gyrus; pre and postcentral gyrus. Corona radiata, head of caudate nucleus, CI, genu, external capsule, thalamus, putamen, insula.
13	Mx	M	66	48	Mixed lesion. Frontal, parietal and temporal lobe.Middle and inferior frontal gyrus; pre and postcentralgyrus. Corona radiata, external capsule, claustrum,putamen, insula

14	Mx	M	29	25	Mixed lesion. Frontal and parietal lobe. Inferior frontal gyrus; precentral gyrus. Insula cortex, external capsule, CI, head of caudate nucleus, genu, putamen, thalamus.
15	Mx	M	47	232	Mixed lesion. Frontal and parietal lobe. Precentral gyrus. Corona radiata, thalamus, putamen, posterior CI, claustrum, external capsule, insula.
16	Mx	M	40	53	Mixed lesion. Frontal, parietal and temporal lobe. Inferior frontal gyrus; pre and postcentral gyrus; supramarginal gyrus. Corona radiata, head of caudate nucleus, CI, genu, putamen, posterior part of external capsule, thalamus, claustrum, Gl. Palidus, trunk of corpus callosum, insula.
17	Mx	M	70	23	Mixed lesion: Frontal and parietal lobe. External capsule, precentral gyrus, putamen, thalamus, CI, genu, insula cortex.
18	Mx	M	54	121	Mixed lesion. Frontal and parietal lobe and the adjacent white matter. Superior, medial, middle and inferior frontal gyrus; pre and postcentral gyrus; supramarginal gyrus. Corona radiata, head of caudate nucleus.
19	Mx	M	61	10	Mixed lesion: cortical and subcortical lesion affecting the insular and temporal lobes, white matter of the parietal and frontal lobe, putamen, CI interna and externa, claustrum, thalamus
20	S	M	65	45	Subcortical lesion. Parietal lobe. White matter of inferior frontal gyrus; pre and postcentral gyrus; supramarginal gyrus. Corona radiata, thalamus, genu, partially CI, external capsule. Also affection the white matter underlying the right insular cortex.

21	S	M	57	122	Subcortical lesion. Corona radiata, external capsule, putamen, posterior CI, thalamus.
22	S	M	47	80	Subcortical lesion. CI, genu, external capsule, claustrum, putamen, head of caudate nucleus, thalamus.
23	S	M	60	130	Subcortical lesion. Parietal lobe. White matter of Precentral gyrus. Corona radiata, anterior CI, putamen, external capsule, thalamus, insula.
24	S	F	72	44	Subcortical lesion. Frontal and parietal lobe. White matter of inferior frontal gyrus; pre and poscentral gyrus; supramarginal gyrus. Multiple nectrotic vesicles. Trunk of corpus callosum, head of caudate nucleus, corona radiata, putamen, external capsule, CI, genu, thalamus.
25	S	F	55	17	Subcortical lesion:frontal and temporal lobe. Body of caudate nucleus, external capsule, putamen, genu, anterior CI, insula cortex.
26	S	M	69	72	Subcortical lesion. Frontal and parietal lobe. Extensive white matter hypodensity of inferior frontal gyrus and precentral gyrus. Corona radiata, head of caudate nucleus, CI, genu, external capsule, putamen, thalamus, insula.
27	S	F	55	45	Subcortical lesion. Corona radiata, head of caudate nucleus, external capsule, CI, genu, putamen, thalamus, globus pallidus, claustrum.
28	S	F	53	30	Subcortical lesion. Corona radiata, external capsule,thalamus, putamen, CI, genu.
29	S	F	53	20	Subcortical lesion. Head of caudate nucleus, CI, genu, putamen, thalamus, corona radiata, external capsule, claustrum.
30	S	M	50	215	Subcortical lesion. Corona radiata, CI, genu, thalamus, external capsule, putamen, claustrum, insula and

					adjacent white matter.
31	S	M	48	45	Subcortical lesion. CI, genu, external capsule,putamen, thalamus, claustrum, head and tail of caudate nucleus, corona radiate, insula.
32	S	M	65	67	Subcortical lesion. Parietal, temporal and insular lobe's white matter, corona radiata, pallidum, capsula interna and externa, thalamus
33	S	M	40	46	Subcortical lesion. Head of caudate nucleus, CI, genu,<br>external capsule, putamen, claustrum, corona radiata.

Mx=mixed lesion (cortical and subcortical), S=subcortical lesion, M=masculine and F= feminine.
