# Supplementary material for: Cortex Integrity Relevance in Muscle Synergies in Severe Chronic Stroke
Source: Front Hum Neurosci. 2014 Sep 23;8:744. doi: 10.3389/fnhum.2014.00744 (PMC4172028; doi:10.3389/fnhum.2014.00744)
Supplement: Supplementary file 2 [file Image1.PDF]

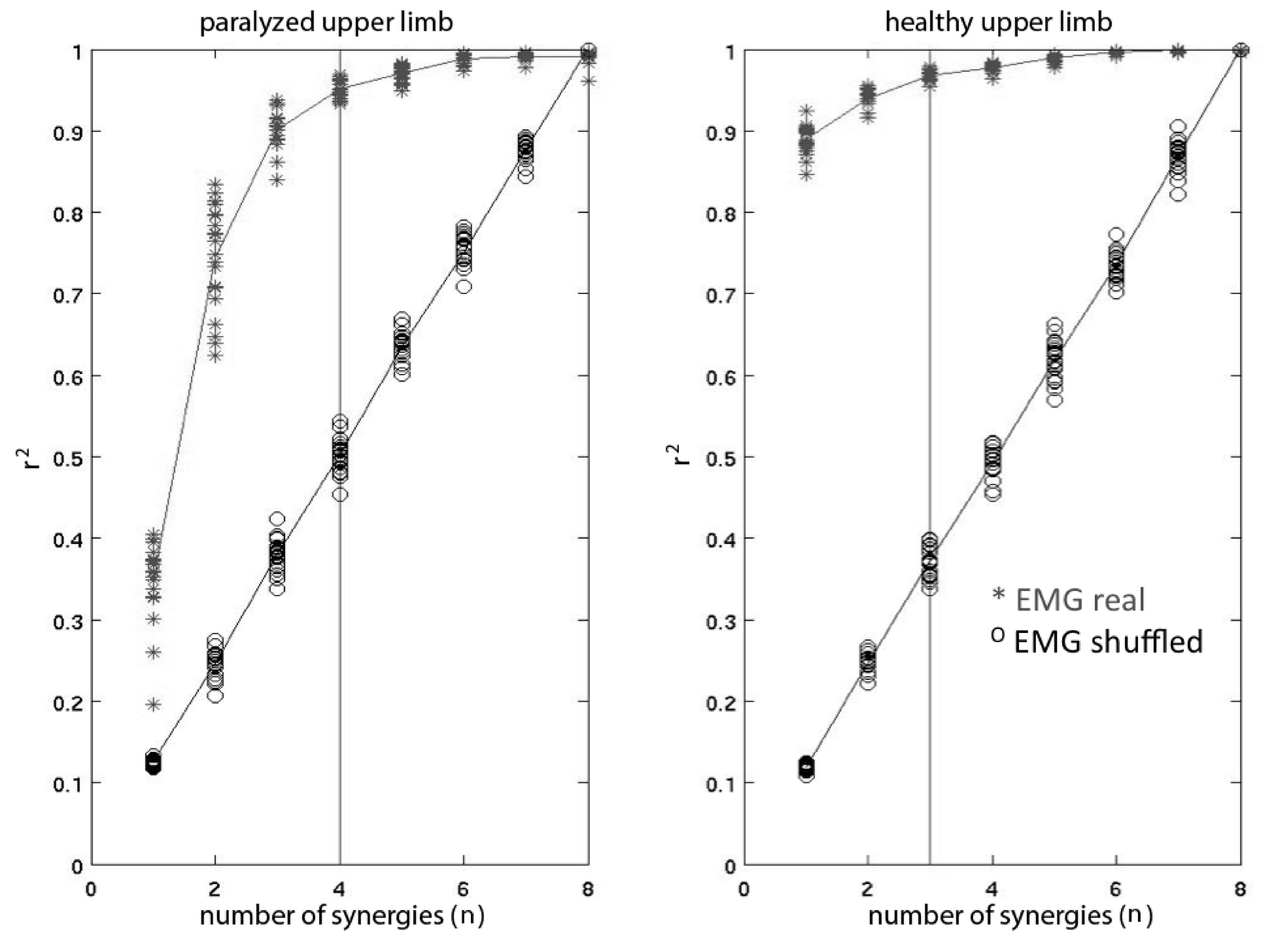
paralyzed upper limb
healthy upper limb
r^2
number of synergies (n)
number of synergies (n)
* EMG real
o EMG shuffled
0
0.1
0.2
0.3
0.4
0.5
0.6
0.7
0.8
0.9
1
0
2
4
6
8

Supplementary Figure 1. Goodness of model fitting in a representative patient. We applied the NMF algorithm (Lee and Seung, 2001) with the number of synergies (n) increasing from one to eight (total number of recording electrodes placed on the relevant muscles). For each patient we tested the goodness of the EMG reconstruction dependent of n (i.e. how similar was to the original EMG activity) by choosing randomly for each n half of the EMG trials for extracting the synergies (W) and the other half for testing them. In this figure we observed the number of synergies needed to explain the EMG data for the paralyzed (right) and healthy (left) upper limbs measured by r^2 values of the regression between reconstructed and real data (*) (being r^2 =1 a perfect reconstruction) and the EMG shuffled data (in time and electrodes) (°) in a 20 fold cross validation (represented by the cloud of points). Furthermore, it is possible to see that the real EMG data (*) contains significant meaningful information compared to the EMG shuffle data (°). The higher the number of synergies included in the calculation the higher the variance of the data explained by the model (i.e. the higher the r^2 value). While the amount of data explained by the shuffled EMG increases linearly with the number of synergies, just few synergies (the most relevant) calculated on the real EMG could explain most of the data. The vertical lines represent the optimal number of synergies selected for the paralyzed (four synergies) and for the healthy upper limb (three synergies) according to the predefined threshold (see *Materials and Methods*).
